# Genome-wide identification, molecular evolution, and expression analysis of auxin response factor (ARF) gene family in *Brachypodium distachyon* L

**DOI:** 10.1186/s12870-018-1559-z

**Published:** 2018-12-06

**Authors:** Nannan Liu, Liwei Dong, Xiong Deng, Dongmiao Liu, Yue Liu, Mengfei Li, Yingkao Hu, Yueming Yan

**Affiliations:** College of Life Science, Capital Normal University, Beijing, 100048 China

**Keywords:** Abiotic stress, *ARF* genes, *Brachypodium distachyon*, Phylogenetic relationships, qRT-PCR

## Abstract

**Background:**

The auxin response factor (*ARF*) gene family is involved in plant development and hormone regulation. Although the *ARF* gene family has been studied in some plant species, its structural features, molecular evolution, and expression profiling in *Brachypodium distachyon* L*.* are still not clear.

**Results:**

Genome-wide analysis identified 19 *ARF* genes in *B. distachyon*. A phylogenetic tree constructed with 182 *ARF* genes from seven plant species revealed three different clades, and the *ARF* genes from within a clade exhibited structural conservation, although certain divergences occurred in different clades. The branch-site model identified some sites where positive selection may have occurred, and functional divergence analysis found more Type II divergence sites than Type I. In particular, both positive selection and functional divergence may have occurred in 241H, 243G, 244 L, 310 T, 340G and 355 T. Subcellular localization prediction and experimental verification indicated that BdARF proteins were present in the nucleus. Transcript expression analysis revealed that *BdARFs* were mainly expressed in the leaf and root tips, stems, and developing seeds. Some *BdARF* genes exhibited significantly upregulated expression under various abiotic stressors. Particularly, *BdARF4* and *BdARF8* were significantly upregulated in response to abiotic stress factors such as salicylic acid and heavy metals.

**Conclusion:**

The *ARF* gene family in *B. distachyon* was highly conserved. Several important amino acid sites were identified where positive selection and functional divergence occurred, and they may play important roles in functional differentiation. *BdARF* genes had clear tissue and organ expression preference and were involved in abiotic stress response, suggesting their roles in plant growth and stress resistance.

**Electronic supplementary material:**

The online version of this article (10.1186/s12870-018-1559-z) contains supplementary material, which is available to authorized users.

## Background

Auxin plays an important role in plant growth and development, including shoot elongation, lateral root formation, vascular tissue differentiation, apical margin patterning, and response to environmental stimuli [1]. The auxin gene family is involved in plant stress response, and includes *Aux/IAA*, *Small Auxin Up RNA* (*SAUR*), and *Gretchen Hagen 3* (*GH3*) [2, 3]. Auxin response factors (ARFs), a critical family of transcription factors in the auxin-mediated pathway, are involved in hormone regulation and plant development [4–6]. They specifically bind the auxin response element (AuxREs) (5’ → 3’TGTCTC) in the promoter region of auxin response genes to regulate gene transcription [1, 7].

ARFs contain three unique domains: a conservative N-terminal DNA-binding domain (DBD), a variable middle transcriptional regulatory region (MR) that functions as an activation domain (AD) or repression domain (RD), and a C-terminal dimerization domain (CTD) [8, 9]. The main function of a DBD, which contains the B3 and auxin_resp domains, is binding the AuxREs of auxin response genes [10]. The type of amino acid in the MR determines whether gene transcription is activated or repressed. An AD is generally rich in glutamine (Q), serine (S), and leucine (L) residues, while a RD is rich in proline (P), serine (S), threonine (T), and glycine (G) residues [7, 9]. A CTD, similar to the PB1 domain of the Aux/IAA protein, includes motifs III and IV that involve protein-protein interactions and mediate the homodimerization of ARFs or the heterodimerization of ARF and Aux/IAA [7, 10, 11]. Plant-specific responses to auxin occur generally through the interaction of ARF and Aux/IAA proteins [12]. When the concentration of auxin is low, the heterodimerization of Aux/IAA and ARF inhibits the transcription activity of ARF, thereby preventing the transcription of related genes. Nevertheless, when the auxin concentration reaches a certain level, the ubiquitin ligase SCF^TIR1/AFB^ subunit ubiquitinates Aux/IAA and degrades it via the 26S proteasome pathway, weakening the inhibitory effect of Aux/IAA on ARF [7, 8].

Since the identification of *ARF* genes in *Arabidopsis thaliana* [13], *ARFs* have been found in about 16 plant species. And their structure, evolution, and expression profiling have been widely investigated in these plants such as *Arabidopsis* [14], rice [15], and maize [16]. ARFs play an important role in plant growth and development. For example, *AtARF2* can regulate floral shedding [17], leaf senescence [18], and seed size [19]; *AtARF3* deficiency causes abnormal floral meristems and reproductive organs [20]; *AtARF5* is associated with the formation of vascular bundles and hypocotyls [21]; *ARF6* and *ARF8* coordinate the transition from immature to mature, fertile flowers [22]; and *AtARF7* and *AtARF19* regulate lateral root formation by activating the *LBD/ASL* gene [23]. In the tomato plant (*Solanum lycopersicum)*, *ARF* genes may influence flower and fruit development [24], and *SlARF4* plays a role in sugar metabolism during fruit development [25]. In rice, *OsARF12* plays a role in regulating phosphate homeostasis [26]. Notably, the *ARF* gene family participates in the response of plants to abiotic stressors [27–30]. For example, many *ARF* genes in *Sorghum bicolor* exhibit significant expression changes in response to salt and drought stress [29]. Some water stress-responsive *GmARFs* in soybean (*Glycine max)* have been identified using quantitative real-time polymerase chain reaction (qRT-PCR) and microarray data [30].

*Brachypodium distachyon* L*.* (2n = 10), the first sequencing member in *Pooideae* [31], is an ideal model plant to study cereals [32]. It has simple growth requirements [32, 33], a small genome, diploid accessions (272 Mb), self-fertility, a short generation time, a small stature but large seeds, and is more closely related to *Triticeae* crops than rice [34]. Although some studies have focused on the *ARF* gene family [14–16], the structural characterization, molecular evolution, expression profiling, and functional properties of the *B. distachyon ARF* family are still not clear. We completed the first comprehensive genome-wide analysis of the *ARF* gene family in *B. distachyon*, and our results provide new insights into the structure, evolution, and function of the plant’s *ARF* family.

## Results

### Genome-wide identification of *ARFs* in *B. distachyon* and other plant species

The 23 and 25 known ARF amino acid sequences from rice and *Arabidopsis,* respectively, were obtained from the RGAP and TAIR databases. These sequences were used as queries for searches in the Phytozome database. If the *E*-value ≤1e–5 of the sequence obtained by BLAST, it is regarded as a candidate sequence. The BLASTP search results were further examined using the online tools SMART [35] and Pfam [36] to confirm the presence of the conserved B3 and Auxin_resp domains, and any redundant or partial sequences were manually removed. The remaining sequences satisfied the amino acid sequences starting from M without N in the CDS and had the whole gene sequences. Ultimately, a total of 19 ARF protein family members from *B. distachyon* and 163 from six other plant species were identified: 20 from *Oryza sativa*, 63 from *Triticum aestivum*, 18 from *Setaria italic*, 25 from *Zea mays*, 21 from *Sorghum bicolor*, and 16 from *Arabidopsis thaliana*. The basic information containing gene name, locus, protein length, intron number, predicted isoelectric point (*pI*), and molecular weight (MW) are shown in Additional file 1: Table S1, and their conserved motifs identified by SMART and Pfam are listed in Additional file 1: Table S2. Generally, *ARFs* encode proteins with 513–1175 amino acids (AA), predicted isoelectric points (*pI*) from 5.45 to 9.18, and molecular weights (MW) between 63.74 and 130.93 kDa.

### Chromosome and subcellular localization of *BdARFs*

**Fig. 1:**
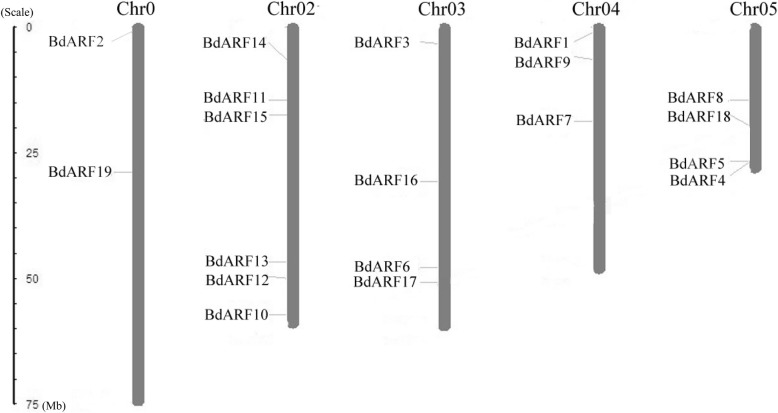
Chromosomal distribution map of *ARF* genes in *B. distachyon*. The chromosome numbers are indicated at the top of each bar while the size of a chromosome is indicated by its relative length. The unit of the left scale is Mb, and the short line indicates the approximate position of the *BdARF* gene on the corresponding chromosome

Using the MapInspect program, all 19 *ARF* genes from *B. distachyon* were mapped to five different chromosomes (Fig. 1): two genes were on chromosome 01 (*BdARF2*:*Bradi1g32547* and *BdARF19*: *Bradi1g33160*), six were on chromosome 02 (*BdARF10*: *Bradi2g59480; BdARF11*: *Bradi2g16610; BdARF12*: *Bradi2g50120; BdARF13*: *Bradi2g46190; BdARF14*: *Bradi2g08120;* and *BdARF15*: *Bradi2g19867*), four on chromosome 03 (*BdARF3*: *Bradi3g04920; BdARF6*: *Bradi3g45880; BdARF16*: *Bradi3g28950* and *BdARF17*: *Bradi3g49320*), three on chromosome 04 (*BdARF1*: *Bradi4g01730; BdARF7*: *Bradi4g17410* and *BdARF9: Bradi4g07470*), and four on chromosome 05 (*BdARF4*: *Bradi5g25767; BdARF5*: *Bradi5g25157; BdARF8*: *Bradi5g10950* and *BdARF18*: *Bradi5g15904*).

**Fig. 2:**
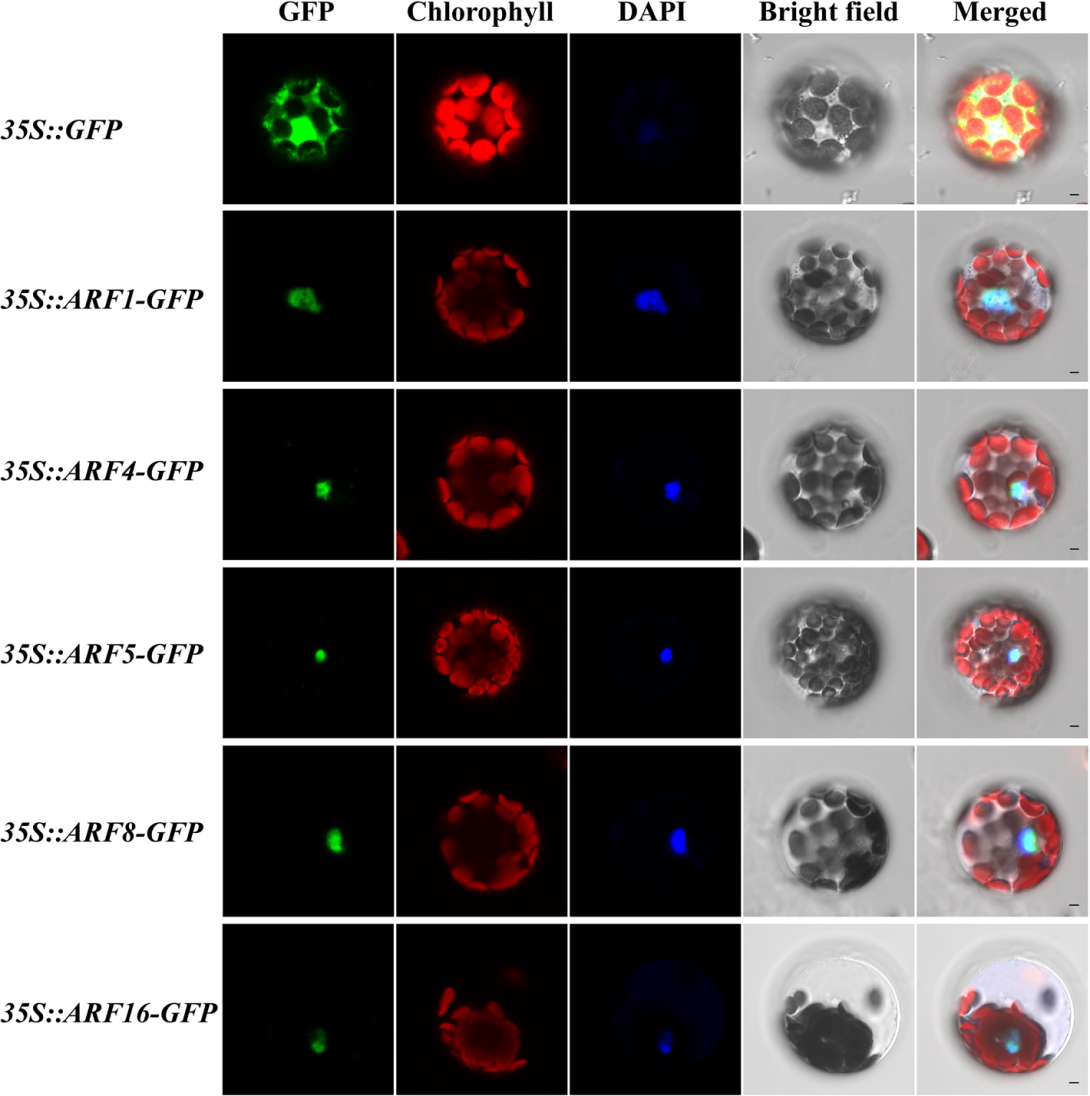
Subcellular localization of five BdARF proteins in *Arabidopsis thaliana* protoplasts. Five proteins included BdARF1, BdARF4, BdARF5, BdARF17 and BdARF19. The localization of the nuclei was detected by 4^′^,6-diamidino-2-phenylindole (DAPI) staining. GFP: GFP fluorescence signal. Green fluorescence indicates the location of BdARFs in the *Arabidopsis* protoplasts; Chlorophyll: chlorophyll autofluorescence signal. Red fluorescent signal indicates the location of chloroplasts in protoplasts; DAPI: Blue fluorescence signal. Blue fluorescence indicates the location of the nucleus stained by DAPI; bright light: field of bright light; Merged: emergence of the GFP fluorescence signal, chlorophyll autofluorescence signal and bright light field; Nagtive: Wild-type (Clo) *Arabidopsis* protoplast cell. Scale bar = 5 μm

The location of proteins encoded by the 19 *ARFs* in *B. distachyon* were all predicted to be located in the nucleus by the five different software programs. To verify these subcellular localization predictions, five *ARF* genes were selected to carry out transient expression with green fluorescent protein (GFP) fusion proteins in *Arabidopsis* suspension culture cells. Confocal laser microscopy was used to perform microexamination. These proteins (BdARF1, BdARF4, BdARF5, BdARF8, and BdARF16) were used to establish recombinant plasmids (*BdARF1::GFP*, *BdARF4::GFP*, *BdARF5::GFP*, *BdARF8::GFP*, and *BdARF16::GFP*) with the 35S promoter. Additional file 1: Table S3 summarizes the primers used. The green fluorescent signals of the five GFP fusion proteins were particularly strong in the nucleus (Fig. 2) and consistent with the predicted results.

### Phylogeny and molecular characterization of *ARFs*

**Fig. 3:**
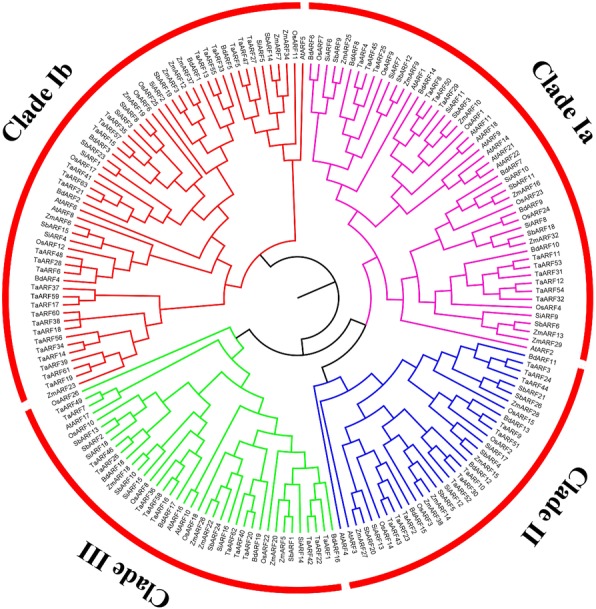
Phylogenetic tree of plant *ARF* gene family. A total of 182 complete protein sequences of the corresponding *ARF* genes obtained from seven plant species were aligned with MUSCLE program, and the phylogenetic tree was constructed based on Bayesian inference using Markov Chain Monte Carlo (MCMC) methods. All ARFs are divided into four branches, each represented by a different color, in which the Ia subfamily is represented by pink, the Ib subfamily is corresponding to red, the II subfamily is represented by blue, and the III subfamily is represented by green. The ARFsfrom *B. distachyon* are indicated by filled yellow rectangle

Multiple sequence alignments of 182 ARF proteins were performed using the Multiple Sequence Comparison by Log-Expectation (MUSCLE) program [37, 38], followed by construction of an unrooted phylogenetic tree using the Markov Chain Monte Carlo (MCMC) method based on Bayesian inference [39]. The 182 ARF proteins were divided into three clades by combining later topology and structure similarity analysis: clade I (including clades Ia and Ib), clade II, and clade III (Fig. 3). Clade Ib was the largest branch with 58 *ARF* gene members, followed by clade Ia (51 members), clade III (39 members), and clade II (34 members). The phylogenetic tree showed that *ARF* genes from monocotyledonous plants such as *B. distachyon* were uniformly distributed in each clade, while those from the dicotyledonous *Arabidopsis* were mainly present in clade Ia.

The exon-intron structures of *ARF* gene family from *B. distachyon* and the other six plant species were analyzed by submitting *ARF* coding sequences (CDSs) and their corresponding gene sequences to Gene Structure Display Server (GSDS) [40]. There were no significant differences in the number of exons among members of the same clade, but noteworthy differences existed among different clades, especially between clade III and other clades (Additional file 2: Figure S1). Clade Ia, clade Ib, and clade II had 12–15 exons, 12–16 exons, and 9–12 exons, respectively. In clade III, all members had 2–5 exons, except for *SbARF1* with 14 exons.

The motif detection software MEME (Multiple Em for Motif Elicitation) [41] was used to perform motif analysis of the ARF protein sequences. A total of 10 conserved motifs were detected from 182 ARFs. The orders and numbers of motifs in a single ARF were shown in Additional file 3: Figure S2, and the sequence composition of each motif was shown in Additional file 4: Figure S3. The number of motifs contained in ARFs generally ranged from 8 to 10. According to the results of the motif detection, the distribution and position of the motifs were relatively conservative among the internal members of the clade. Interestingly, almost all members of clade II had 8 motifs, significantly different from other clades with 10–11 motifs.

### Functional divergence and adaptive selection

To explore whether the amino acid substitutions lead to functional divergence, the Type I and II functional divergences of the gene cluster in *ARF* family were estimated using the DIVERGE v2.0 program [42, 43] (Table 1). The results revealed that the Type I functional divergence coefficient (θI) between any two clades of ARF protein ranged from 0.209 to 0.733, which was significantly greater than 0. The LRT value reached a significant difference (*p* < 0.5), indicating the possible presence of Type I divergence sites during the evolution between the clades of plant ARF proteins. Similarly, the Type II functional divergence coefficient (θII) ranged from − 0.161 to 0.113, which is also significantly greater than 0, indicating that Type II functional divergence sites may be also present.

**Table 1:** Functional divergence between clades of the *ARF* gene family

Group1	Group2	Type I			Type II	
		θI ± s.e.	LRT	Qk > 0.95	θII ± s.e.	Qk > 0.95
Ia	Ib	0.209 ± 0.075	7.078931**	0	−0.161 ± 0.189	0
Ia	II	0.422 ± 0.049	64.383411**	8	0.041 ± 0.191	6
Ia	III	0.631 ± 0.049	109.075555**	12	0.081 ± 0.227	41
Ib	II	0.612 ± 0.073	54.760541**	2	−0.048 ± 0.181	3
Ib	III	0.733 ± 0.071	69.986229**	8	0.113 ± 0.211	27
II	III	0.485 ± 0.070	46.482589**	3	−0.055 ± 0.251	15

Note: θI and θII, the coefficients of Type-I and Type-II functional divergence; LRT, Likelihood Ratio Statistic,* and ** representative *p* < 0.05 and *p* < 0.01, respectively; Qk, posterior probability

In accordance with Yang et al. [44], the posterior probability (Qk) of divergence for each amino acid site was calculated to identify key sites related to functional divergence between every two clades. Large Qk values indicate a high probability of functional divergence between two clades [42]. Type I and II functional divergence residues with Qk ≤ 0.95 were excluded to reduce false positives. The results revealed that Type I and II functional divergence sites existed between every two clades, and variable numbers of Type I functional divergence sites (ds) were found between pairs of clades: clade Ia and Ib (0 ds), Ia and II (8 ds), Ia and III (12 ds), Ib and II (2 ds), Ib and III (8 ds), and II and III (3 ds). In contrast, Type II sites were much more common than Type I sites (Table 1). The specific Type I and II functional divergence sites are shown in Additional file 1: Table S4. Particularly, 8 amino acid sites underwent both the Type I and II functional divergence, indicating that their evolutionary rates and physicochemical properties were altered (Additional file 5: Figure S4).

To assume variable selective pressure among sites, a site-specific model was applied to the *ARF* gene families in the seven selected plant species. Two pairs of models, M0 (one scale) and M3 (discrete), as well as M7 (beta) and M8 (beta and ω) [45], were applied in this analysis (Additional file 1: Table S5). No amino acid sites were identified as being under positive selection, indicating that the *ARF* gene family is not under selective pressure. The branch site model of *ARF* gene family was further analyzed using site-specific analysis (Table 2). Seven positive selection sites in clade Ia were identified, including four critical positive selection sites (340G, 355 T, 356 T and 358P, *p* < 0.01). Meanwhile, 44 and 20 positive selection sites were present in clade II and clade III respectively, including 34 critical sites in clade II (232S, 250H, 259 T, *p* < 0.05; 230 M, 238D, 239S, 240 M, 241H, 243G, 244 L, 246A, 247A, 255 N, 275 L, 276A, 279 V, 282 V, 287 V, 289 V, 295 M, 308 M, 310 T, 312 T, 314I, 337S, 338 T, 340G, 343Q, 344P, 345R, 353P, 354 L, 358P, and 371P, *p* < 0.01) and 12 critical sites in clade III (253A, 254 T, 286R, *p* < 0.05; 204D, 230 M, 231P, 232S, 233S, 236S, 237S, 246A, and 144G, *p* < 0.01). Interestingly, clade Ib had no positive selection sites, implying that clade is under neutral or negative selection, while clades Ia, II and III may undergo strong positive selection. In particular, six sites (241H, 243G, 244 L, 310 T, 340G and 355 T) experienced two types of functional divergences and positive selection pressure (Additional file 5: Figure S4).

**Table 2:** Parameters estimation and likelihood ratio tests of *ARF* genes for the branch-site models

Clade	Model	np^a^	lnL	2△lnL	Positive selected sites^b^
Ia	Model A-null	365	−30,653.840481		Not allowed
	Model A	366	−30,653.840481	0	169D, 339 A, 340 G**, 355 T**, 356 T**, 358 P**, 360 Y
Ib	Model A-null	365	−30,683.021342		Not allowed
	Model A	366	−30,683.021342	0	None
II	Model A-null	365	−30,601.447106		Not allowed
	Model A	366	−30,601.447106	0	229 I, 230 M**, 231 P, 232 S*, 236 S, 238 D**, 239 S**, 240 M**, 241 H**, 243 G**, 244 L**, 246 A**, 247 A**, 250 H*, 252 A, 255 N**, 259 T*, 266 S, 273 I, 275 L**, 276 A**, 279 V**, 281 S, 282 V**, 283 Y, 287 V**, 289 V**, 291 M, 295 M**, 297 F, 308 M**, 310 T**, 312 T*, 314 I**, 337 S**, 338 T**, 340 G**, 343 Q*, 344 P**, 345 R*, 353 P*, 354 L**, 358 P**, 371 P**
III	Model A-null	365	−30,624.183120		Not allowed
	Model A	366	−30,624.183120	0	193 V, 204 D**,213 N, 228 T, 230 M**, 231 P**, 232 S**, 233 S**, 236 S**, 237 S**, 241 H, 246 A**, 250 H, 698 A, 700 A*, 701 T*,745 Y,749 R*, 752 V, 832 G**

Note: *p* < 0.05 and *p* < 0.01 were marked by* and **, respectively. All sites are located on reference amino acid sequence BdARF1 according to the multiple sequence alignment result. Sites are highlighted in red subjected to functional divergence and positive selection

### Promoter analysis of *ARF* gene family

Promoter regions contain *cis*-acting elements that regulate the expression of genes. The *cis*-acting elements in the 1500 bp region upstream of the *ARF* gene family were investigated by PlantCARE online tool [46]. In total, seven types of elements were identified: hormone responsive, environmental stress related, promoter related, site binding related, light responsive, developmental related elements, and others. Among them, photoperiod, developmental regulation, hormonal response, and environmental response were the key physiological processes closely related to the regulatory elements (Additional file 1: Table S6).

We analyzed the hormone responsive elements present in *ARF* gene family of *B. distachyon*, including P-box [47], GARE-motif [48], TCA-element [49], ABRE [50], TGACG-motif, and CGTCA-motif. Among them, ABRE, TGACG-motif, and CGTCA-motif were abundant, and the average copy numbers were 2.737, 1.789, and 1.789, respectively. These elements were mainly related to abscisic acid (ABA) and methyl jasmonic acid (MEJA) stresses.

Additionally, the *cis*-elements responding to environmental stressors were attracted and identified in *B. distachyon*, including HSE [51], LTR, GC-motif and ARE [52], Box-W1, WUN-motif, and TC-rich [52]. Notably, GC-motif and ARE elements, involved in the regulation of gene expression in the absence of oxygen stress, were found to be abundant (1.000 and 1.474 copies). Development related elements, such as GCN4-motif, and CAT-box, were identified, which were relatively abundant (Additional file 1: Table S6) and associated with endosperm development and meristem growth, but their abundance did not differ significantly between monocotyledonous and dicotyledonous plants. Meanwhile, photoreactive element *Sp1* is absent in the dicotyledonous *Arabidopsis thaliana*, but the abundance in *B. distachyon* (1.316 copy) is relatively high. This is similar to other monocotyledonous cereals, suggesting that *ARF* genes may play a role in photosynthesis or carbohydrate synthesis.

### Three-dimensional structure prediction of BdARF proteins and identification of critical amino acid sites

**Fig. 4:**
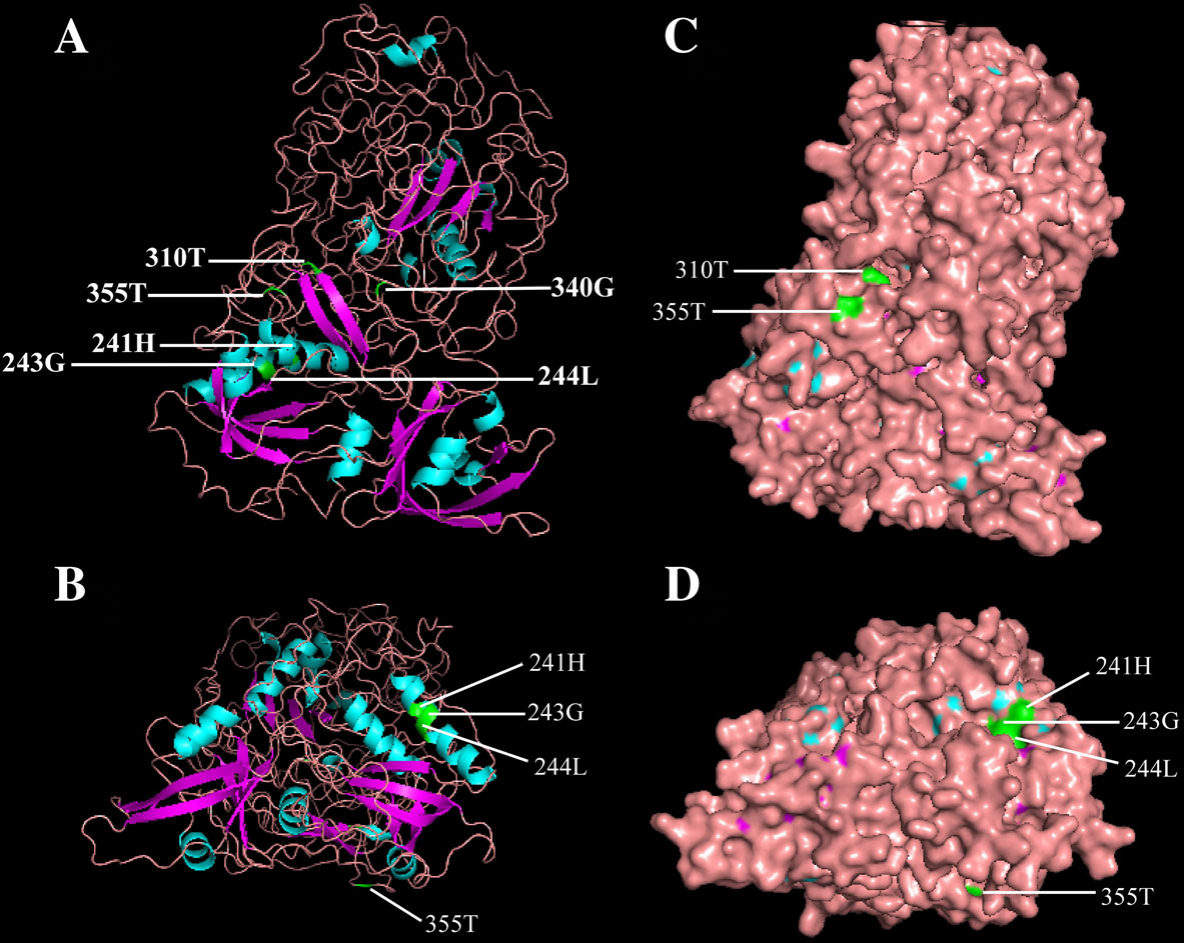
Three-dimensional structure of *B. distachyon* ARF protein BdARF1. **a**. Schematic diagram of 3D structure of BdARF1. **b** Schematic diagram of 3D structure of BdARF1. It is obtained by (**a**) rotating 180 degrees clockwise and then 90 degrees upward. **c** Surface representation of BdARF1 corresponding to (**a**). **d** Surface representation of BdARF1 corresponding to (**b**). The precise positions of six critical amino acids were identified among Type I and Type II functional divergence and positive selection in the 3D structure. Five unique amino acid sites are shown on the surface of the 3D structure. In the figure, helix is represented by light blue, purple represents sheet, pink represents loop, and the six key amino acid positions are indicated by green. The amino acids represented by the six key positions and their positions in the protein sequence are labeled

The three-dimensional structures of 19 BdARFs from *B. distachyon* were visually predicted by searching the PHYRE2 database and using Pymol software [53]. All BdARF proteins had similar structural features; a representative BdARF1 is shown in Figure 4. Six key sites (688H, 690G, 691 L, 774, 839G and 856 T) identified by positive selection and functional divergence are marked in green in the 3D structure (Fig. 4a and b). Three sites (241H, 243G and 244 L) were located on helix, two (340 T and 355 T) on the loop and one (310 T) on the sheet. Interestingly, 5 sites (241H, 243G, 244 L, 310 T and 355 T) exist on the surface of the 3D structure (Fig. 4c and d).

### Protein interaction analysis of BdARFs

The study of protein-protein interactions (PPI) facilitates understanding of gene function [54]. Therefore, to better understand whether ARF exerts biological functions through protein interaction, the STRING database was used to construct the PPI network for BdARFs [55]. The PPI networks and potential substrates were extracted from the whole interaction network and reconstructed using the software Cytoscape (version 3.0.2). A total of 10 BdARFs (none from clade II) and 5 Aux/IAA proteins were identified in the PPI network (Additional file 6: Figure S5).

### Expression of *B. distachyon ARF* genes in different tissues and organs

The expression profiles of the 19 *B. distachyon ARF* genes from different clades in six tissues and organs were analyzed by qRT-PCR, including root, stem, leaf, root tip, leaf tip, and developing seeds at 15 days post-anthesis (DPA). The primer sequences for qRT-PCR assays are listed in Additional file 1: Table S7. The optimal parameters yielded a correlation coefficient (r^2^) of 0.994–0.999 and PCR amplification efficiency (E) of 90–110% (Additional file 7: Figure S6, Additional file 8: Figure S7).

**Fig. 5:**
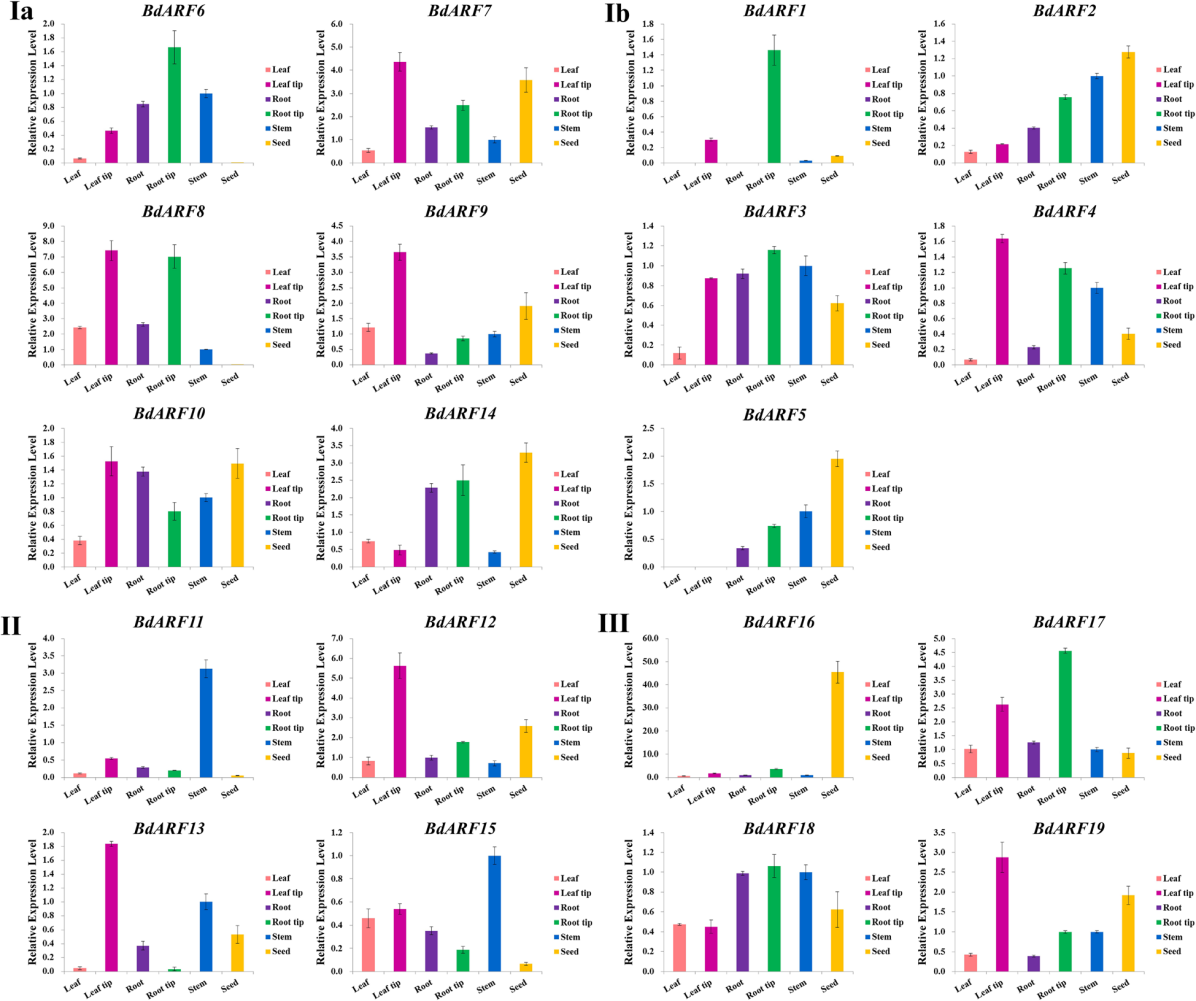
The tissue and organ expression patterns of 19 *B. distachyon ARF* genes. The expression profiles of 19 *B. distachyon ARF* genes in different tissues and organs, including leaf, leaf tip, root, root tip, stem, and seed (15 DPA). Different tissues and organs are represented by different colors: orange for the leaf, red for the leaf tip, purple for the root, green for root tip, blue for stem, and yellow for seed. 19 *BdARFs* are sorted according to their clade. And the ordinate represents the expression level, and the abscissa shows different tissues and organs

As shown in Fig. 5, *BdARFs* generally had high expression levels in leaf and root tips, stems, and developing seeds. The *BdARF* genes from different clades also had expression differences. For clade I, *BdARFs* were mainly expressed in leaf and root tips or seeds. Six *BdARF* genes (*BdARF6*, *BdARF7*, *BdARF8*, *BdARF9*, *BdARF10*, and *BdARF14*) from clade Ia had high levels of expression in the leaf/root tips. Except for *BdARF6* and *BdARF8,* the other four genes were also highly expressed in the seeds. This is consistent with the promoter analysis indicating that *BdARF6* and *BdARF8* have almost no *cis*-acting elements associated with endosperm development. The *BdARFs* from clade Ib also had similar expression preference. For example, *BdARF1* was abundantly expressed in root tips, but was not expressed in roots and leaves. *BdARF5* was expressed in all tissues and organs, except leaves, and its expression level was highest in developing seeds. *BdARFs* from clade II were mainly expressed in stem and leaf tips, of which *BdARF11* and *BdARF15* were mainly expressed in the stems, while *BdARF12* and *BdARF13* were mainly expressed at the leaf tips. *BdARFs* from clade III were mainly expressed in the leaf and root tips and developing seeds, of which *BdARF16* had an extremely high expression level in the seeds (Fig. 5).

### Expression profiling of *B. distachyon ARFs* in response to various abiotic stressors

**Fig. 6:**
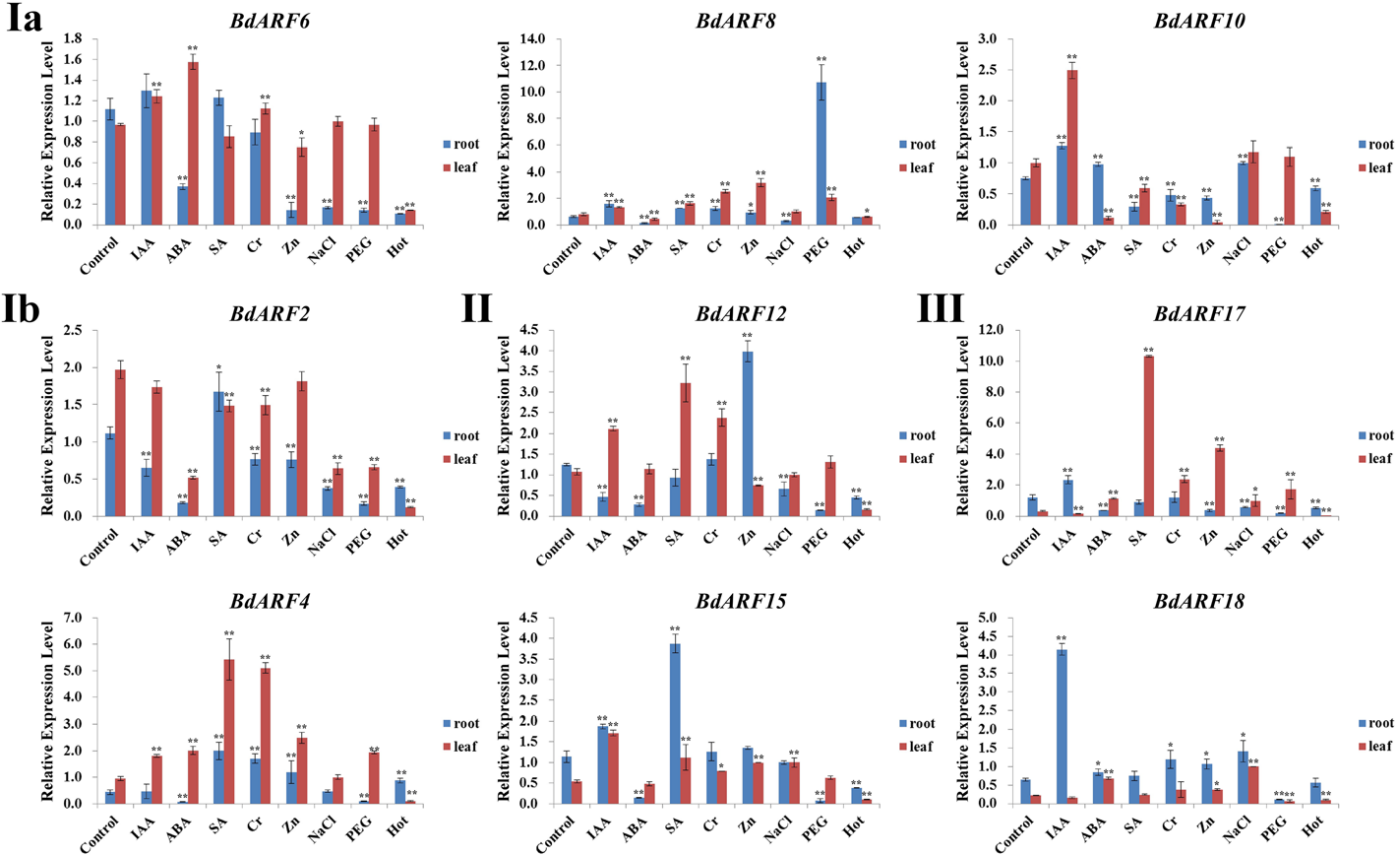
Expression patterns of *B. distachyon ARF* genes under various abiotic stresses. The expression profiles of nine representative *B. distachyon ARF* genes under various abiotic stresses, including IAA, ABA, SA, Cr^3+^, Zn^2+^, NaCl, PEG and Hot (42 °C). IAA, indole-3-acetic acid; ABA, abscisic acid; SA, salicylic acid; PEG, polyethylene glycol. The leaf and root are separately analyzed, red color bar presents leaf, sky blue color bar presents root. Statistically significant differences between control group and treatment group were calculated by an independent Student’s *t*-tests: *p < 0.05, **p < 0.01. 9 *BdARFs* are sorted according to their clade (Clade **Ia**: BdARF6, BdARF8, BdARF10; Clade **Ib**: BdARF2, BdARF4; Clade **II**: BdARF12, BdARF15; Clade **III**: BdARF17, BdARF18). And the ordinate represents the expression level, and the abscissa shows different tissues and organs

Nine representative *BdARFs* from the three clades were selected to further detect their expression patterns in both roots and leaves under different abiotic stressors (Fig. 6). These genes included *BdARF6*, *BdARF8,* and *BdARF10* from clade Ia, *BdARF2* and *BdARF4* from clade Ib, *BdARF12* and *BdARF15* from clade II, and *BdARF17* and *BdARF18* from clade III. The abiotic stressors used were osmotic (NaCl and polyethylene glycol (PEG)), heat (42 °C), heavy metals (Zn^2+^ and Cr^3+^), and phytohormones ABA, indole-3-acetic acid (IAA), and salicylic acid (SA). The primer sequences, optimal parameters, and PCR amplification efficiency are listed in Additional file 1: Table S7, Additional file 7: Figures S6 and Additional file 8: Figure S7, respectively.

In general, most of the nine *BdARFs* exhibited significantly upregulated expression in both roots and leaves in response to single and multiple abiotic stress treatments. Meanwhile, transcriptional expression differences were observed between *BdARF* members from different clades, including significant upregulation of *BdARF8* from clade Ia under IAA, SA, Cr^3+^, Zn^2+^, and PEG stressors; *BdARF10* from the same clade under IAA stress; *BdARF4* from clade Ib under SA, Cr^3+^, and Zn^2+^ stressors; *BdARF15* from clade II under IAA and SA stressors; and *BdARF18* from clade III under ABA, Zn^2+^, and NaCl stressors (Fig. 6).

The *BdARFs* between roots and leaves also exhibited clear differences in expression under the abiotic stressors. In roots, almost all *BdARF* genes under IAA treatment were significantly upregulated, except for *BdARF2* and *BdARF12,* which were significantly downregulated. In contrast, only *BdARF10* and *BdARF18* were upregulated and the others were downregulated under ABA treatment. The SA treatment significantly upregulated *BdARF2*, *BdARF4*, *BdARF8*, and *BdARF15*. Under heavy metal (Zn^2+^ and Cr^3+^) stressors, *BdARF4*, *BdARF8,* and *BdARF18* were significantly upregulated. When plants were suffering from osmotic stress, *BdARF* genes were more sensitive in roots than leaves, and generally exhibited significant downregulation after PEG and NaCl treatments. For heat stress, all genes were downregulated, except *BdARF4* was significantly upregulated.

In leaves, *BdARFs* were generally significantly upregulated from hormone treatments, including all *BdARF* genes except *BdARF2*, *BdARF17,* and *BdARF18* under IAA stress, four genes (*BdARF6*, *BdARF4*, *BdARF17,* and *BdARF18*) under ABA stress, and five genes (*BdARF4*, *BdARF8*, *BdARF12*, *BdARF15,* and *BdARF17*) under SA stress. When subjected to heavy metal (Zn^2+^ and Cr^3+^) treatments, most of the nine *BdARFs* were upregulated, except two genes (*BdARF2* and *BdARF10*) under Cr^3+^ stress and three genes (*BdARF6*, *BdARF10* and *BdARF12*) under Zn^2+^ stress were significantly downregulated. Under osmotic stress, *BdARF15*, *BdARF17,* and *BdARF18* were significantly upregulated under NaCl stress and *BdARF4*, *BdARF8,* and *BdARF17* were significantly upregulated under PEG stress. However, all *BdARFs* were downregulated in response to heat stress (Fig. 6).

**Fig. 7:**
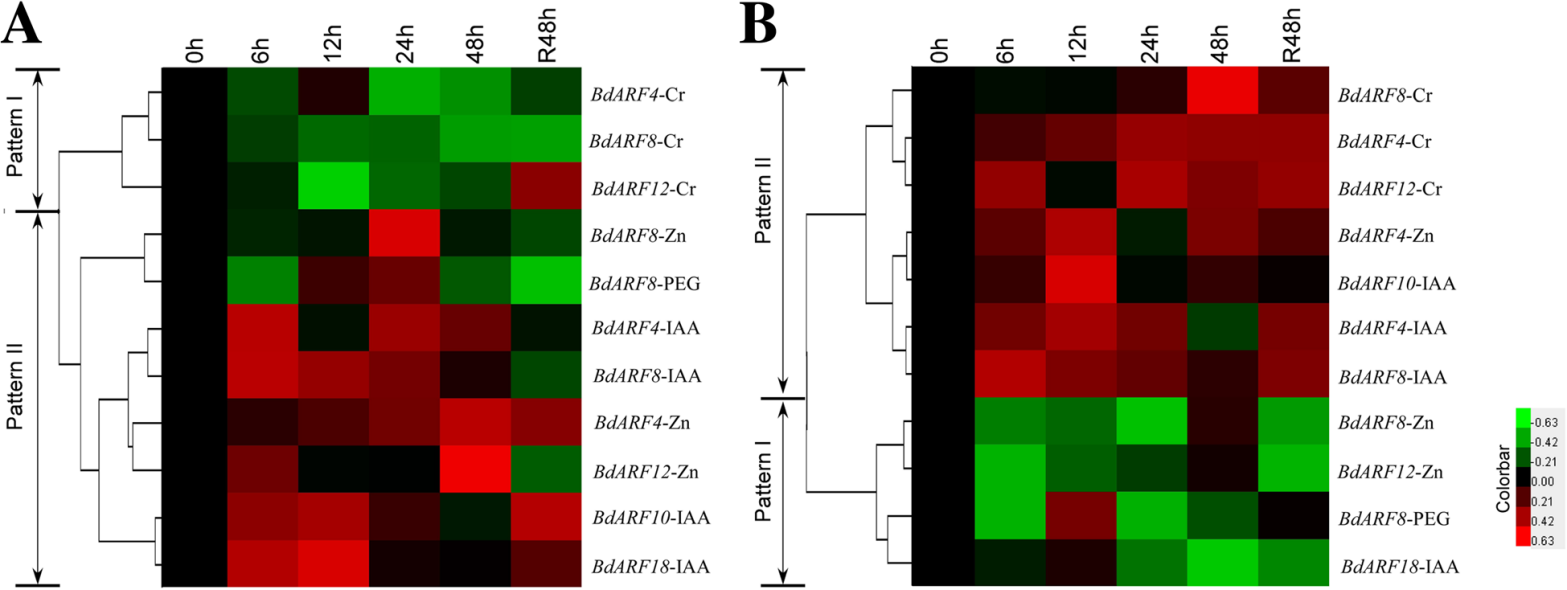
Dynamic expression profiles of *B. distachyon ARF* genes under abiotic stress. **a** *BdARF* genes expression profiles in the root. **b** *BdARF* genes expression profiles in the leaf. Samples were harvested at 0, 6, 12, 24, 48 h, and recovery 48 h. The expression levels of *ARF* genes at 0 h were defined as 1.0 in both organs. The dynamic expression pattern is divided into two types as a whole. Pattern I: The overall expression level is lower than 0 h. Pattern II: The overall expression level is higher than 0 h

Our results revealed that five *BdARF* genes (*BdARF4*, *BdARF8*, *BdARF10*, *BdARF12,* and *BdARF18*) in roots and leaves generally displayed a significantly upregulated expression under Cr^3+^, Zn^2+^, PEG, and IAA treatments. Thus, the dynamic expression patterns of these *BdARFs* at six time points (0, 6, 12, 24, and 48 h, and recovery for 48 h) under four stress treatments (Cr^3+^, Zn^2+^, PEG, and IAA) were further investigated (Fig. 7). The results revealed that *BdARFs* generally exhibited a distinct expression pattern of upregulation in leaves and downregulation in roots after stress treatments. Most *BdARFs* in roots were upregulated at 6, 12, and 24 h after treatments and downregulated at 48 h and after 48 h of recovery (Fig. 7a). In leaves, *BdARFs* were upregulated at 6, 12, 24, and 48 h after stress treatments and at 48 h of recovery (Fig. 7b). Interestingly, the expression patterns of *BdARF4*, *BdARF8,* and *BdARF12* were upregulated in both leaves and roots in response to IAA stress, but exhibited an opposite trend in roots (downregulation) and leaves (upregulation) under Cr^3 +^ stress.

## Discussion

### Molecular characterization and evolution of the *ARF* gene family in *B. distachyon*

Genome-wide analysis indicated that 19 *BdARFs* from *B. distachyon* and 163 from six other plant species were divided into three clades (Fig. 3). The *ARFs* within a clade have similar intron-exon structure characteristics, suggesting the conservation of *ARF* structures within clades (Additional file 2: Figure S1). Most of the *ARFs* motifs are concentrated in 8–10 (Additional file 3: Figure S2 and Additional file 4: Figure S3), demonstrating the evolutionary conservation of *ARFs* among plant species. However, almost all of the clade II members lost two motifs, probably due to the influence of selection pressure in the evolutionary process. Further protein predictive analysis revealed that ARF can perform biological functions by forming homodimers or heterodimers, consistent with previous reports [10, 11, 56]. The clade II has no members involved in the protein interaction network (Additional file 6: Figure S5), possibly due to the lack of these two important motifs.

In a gene family, new genes produced by replication may either be retained due to the evolution of new functions, or they may be lost [57]. Generally, replicative genes are not under selective pressure in the early stages of evolution or do not exhibit the characteristics that usually appear under positive selection pressure. In the evolution of special functions, selection pressure tends to be negative because each gene has a fixed function [58, 59]. In this study, a site-specific model did not detect any positive selection sites (Additional file 1: Table S5), and the maize *ARF* gene family was found to be under negative selection [60]. Therefore, we speculate that the ancient plant ARF proteins have undergone negative selection to maintain their function.

Amino acid site mutations occur frequently and accumulate mass variations such that duplicate genes have diverged in function [61–64]. We found that functional disproportionation occurred between each pair of clades, and the number of Type II disproportionation sites was much greater than the Type I (Additional file 1: Table S4). The functional divergences between clades are mainly attributed to changes in the physicochemical properties of amino acids and changes in the rate of evolution [42]. Furthermore, 8 key amino acid sites have both Type I and Type II functional divergences. The evolutionary rate and physiochemical properties of these sites have changed, indicating that these sites are important for the functional differentiation of the ARF protein family. Particularly, six critical amino acid sites underwent both functional divergence and positive selection pressure, which could play key roles in the domain differentiation (Additional file 5: Figure S4).

### Expression and functions of *B. distachyon ARFs* in response to abiotic stressors

Auxin plays an important role in the response to abiotic stressors, and ARF is an important transcription factor in the auxin signaling pathway [65]. The promoter region of the *ARF* contains a large number of *cis*-acting elements associated with abiotic stress, suggesting that *ARF* may participate in stress defense.

The activity of ARFs is regulated by the concentration of auxin [8], so we focused on the effect of exogenous hormones on *ARF* expression. Almost all *BdARFs* were significantly upregulated in *B. distachyon* seedlings, roots, and leaves when subjected to IAA treatment. This phenomenon may be due to the fact that exogenous IAA affects the homeostasis of auxin and promotes the upregulation of transport inhibitor resistant 1 (TIR1), which has an F-box domain that binds to the SCF ubiquitin ligase to form the SCF^TIR1^ complex [66]. When Aux/IAA binds to the SCF^TIR1^ complex, it is ubiquitinated and subsequently degraded by the proteasome [4, 67]. Subsequently, ARF protein is released and accumulates due to the degradation of Aux/IAA, which activates or inhibits the expression of downstream genes [68]. As a critical plant hormone, ABA participates in the response to a wide range of abiotic stressors and regulates stress tolerance to cope with environmental stressors [69]. Plants can sense changes in the external environmental in a variety of ways, one of which is changes in auxin concentration [70]. When a stressor causes an increase in auxin, the plant activates ABA and other pathways [71], and promotes the expression of stress-defense genes, such as C-repeat/dehydration-responsive element-binding factors (CBFs) or responsive to dehydration (RDs) genes [72]. Meanwhile, a large number of *GH3* family genes are induced by promoting the expression of ARF genes to negatively regulate auxin levels [72]. In the present study, we found that exogenous ABA and SA usually upregulated *BdARFs* in leaves (Fig. 6). Therefore, *BdARFs* could be induced by ABA and SA, which activate the downstream genes to respond to phytohormone stress [15].

Heavy metal contamination is an increasingly serious global problem. An increase of reactive oxygen species (ROS) is a common phenomenon when plants are exposed to heavy metal stress [73, 74]. Along with increased ROS, plants cope with stress through the signaling pathway mediated by mitogen-activated protein kinase (MAPK) [75, 76]. The activated MAPK pathway may fight stress primarily by inducing the expression of some protective genes and activating the expression of repressive A*RFs* [77]. Additionally, ROS can influence the ubiquitin degradation pathway associated with ARF by inhibiting TIR1, further affecting endogenous auxin levels [70].

Under NaCl and PEG treatments, most *BdARFs* were inhibited, but *BdARF8*, *BdARF10,* and *BdARF18* were significantly upregulated (Fig. 6), indicating that they are involved in osmotic stress response. A previous study found that *SlARF8A* and *SlARF10A* were upregulated in response to salt and drought stress in tomato plants [78]. Furthermore, *AtARF8* in Arabidopsis may be involved in auxin homeostasis by affecting the growth habits of hypocotyls and roots [79]. *AtARF10* influenced the formation of the root, implying it plays an important role in coping with stress [80]. Additionally, under salt and drought stress, plants activate *GH3* through the expression of *ARFs* and ABA pathway to maintain auxin homeostasis and activate relevant stress response genes to weaken or eliminate the effects of stress [72]. Our results suggest that only the *BdARF4* gene was significantly upregulated in roots under heat stress. To date, the mechanism of plant *ARFs* defense against heat stress is not clear. Numerous studies have found that ABA/SA and Ca^2+^, which represent ABA-dependent and calcium-dependent protein kinase (CDPK) signaling pathways, are involved in plant heat stress response [81–83]. *BdARF4* may participate in these signaling pathways to resist heat stress. Moreover, although plants maintain auxin homeostasis through an ARF-associated ubiquitin degradation pathway and an ABA pathway to cope with hormonal, osmotic, and heat stressors, the stress can also induce the accumulation of ROS and crosstalk with the ABA and other pathways to deal with complex abiotic stress [70, 84–86].

## Conclusions

Genome-wide analysis identified 19 *BdARFs* in *B. distachyon* and 163 from six other plant species, which were divided into four clades. The intron-exon structure analysis revealed an evolutionarily conserved *ARF* gene family. The functional divergence between clades was mainly attributed to changes in the physicochemical properties of amino acids, followed by changes in the rate of evolution. In particular, functional divergence and positive selection occurred simultaneously at six sites (241H, 243G, 244 L, 310 T, 340G and 355 T), suggesting their important roles in domain differentiation. BdARFs were located in the nucleus by subcellular localization prediction and empirical evidence. qRT-PCR analysis revealed that the expression of *BdARFs* had a tissue and organ expression preference, generally with high expression levels in leaf and root tips, stems, and developing seeds. Meanwhile, some *BdARFs* were significantly upregulated in response to abiotic stressors, indicating their involvement in stress resistance. Our results provide new evidence for further understanding the structure, evolution, and function of the plant *ARF* gene family.

## Methods

### Genome-wide identification of *ARF* genes in *Brachypodium distachyon* L

*ARF* genes from *Brachypodium distachyon* and other six plant species that represent plant lineages of monocots and dicots were identified, including *Setaria italic*, *Oryza sativa*, *Sorghum bicolor*, *Zea mays*, *Triticum aestivum* and *Arabidopsis thaliana*. ARF amino acid sequences from *rice* and *Arabidopsis* were acquired from the RGAP (http://rice.plantbiology.msu.edu/) and TAIR (http://www.arabidopsis.org/) databases, respectively. The possible ARFs in the respective plant species were retrieved in the plant database (http://www.phytozome.org; http:// wheat-urgi.versailles.inra.fr/Seq-Repository) [87] by BLASTP analysis using ARFs from *rice* and *Arabidopsis* as a query. All identified ARFs were further validated by a conserved domain search using SMART (http://smart.embl-heidelberg.de/) [35] and PFAM (http://pfam.xfam.org/) [36] databases, whose E values were less or equal to 1E-5, consequently the redundant and partial sequences were removed manually.

### Chromosomal locations, subcellular localization and phylogenetic analysis

The location of *ARF* genes on *B. distachyon* chromosomes obtained from Phytozome database was mapped by MapInspect program and manually modified.

The subcellular localization of ARFs was predicted according to the integration of prediction results of FUEL-mLoc Server (http://bioinfo.eie.polyu.edu.hk/FUEL-mLoc/) [88], WoLF PSORT (http://www.genscript.com/wolf-psort.html) [89], CELLO version 2.5 (http://cello.life.nctu.edu.tw/) [90], Plant-mPLoc (http://www.csbio.sjtu.edu.cn/bioinf/plant-multi/) [91] and UniProtKB (http://www.uniprot.org/). To verify the subcellular localization prediction, the full-length coding sequences of *ARFs* without stop codon were cloned into the green fluorescent protein (GFP) vector to carry out subcellular localization. This recombined plasmid was transiently transformed into *Arabidopsis* mesophyll protoplasts by PEG-mediated transformation [92]. After an overnight incubation at 26 °C in the dark, GFP signal was detected by a Zeiss LSM 780 fluorescence confocal microscopy.

Phylogenetic trees were constructed based on Bayesian inference using Markov Chain Monte Carlo (MCMC) method [39]. Multiple sequence alignment using full protein sequences were performed based on MUSCLE program (http://www.ebi.ac.uk/Tools/msa/muscle/) [37, 38].

### Protein properties and sequence analysis

pI/MW of ARFs was determined by the Compute pI/MW tool in ExPASy proteomics server database (http://web.expasy.org/compute_pi/) [93]. The exon-intron structure of ARF genes was derived from the online Gene Structure Display Server v2.0 (GSDS: http://gsds.cbi.pku.edu.cn) with coding sequences (CDS) and genomic sequences [40]. The MEME program (Multiple Em for Motif Elicitation v 4.10.2 (http://meme-suite.org/tools/meme) [41] was employed to identify conserved motifs in the candidate ARF protein sequences. The MEME program was run locally and the parameters were set to a maximum of 10 motifs.

The 1500 bp upstream region of the *ARF* member region was used as the promoter distribution region, and the promoter sequence of the *ARF* member was obtained from the Phytozome (www.phytozome.net) database. The resulting promoter sequences were submitted for promoter *cis*-element analysis in the PlantCARE database (http://bioinformatics.psb.ugent.be/webtools/plantcare/html/) [46].

### Positive selection and functional divergence analyses

This study used the maximum likelihood method to test positive selection, two models in the CODEML program in the PAML package [45, 94, 95]: site model and branch-site model were used to test whether members of the ARF protein family were positively selected during evolution. Using DIVERGE v2.0 software package combined with posterior probability analysis to analyze the function disproportionation of type I and type II between different subfamilies of the ARF family [42, 43, 96].

### Three-dimensional structure visualization of BdARF protein

The 3D structure of BdARF protein was constructed using the online software PHYRE2 (http://www.sbg.bio.ic.ac.uk/phyre2/html/page.cgi?id=index) [53]. Then, editing was performed by Pymol software (version 1.7.4 Schrödinger, LLC., http://pymol.org/) to visualize the three-dimensional structure of BdARF protein and to label the screened important amino acid sites on the 3D structure map.

### Prediction of ARFs interaction with related proteins

The protein sequences of the ARFs were collected by BLAST analysis with the NCBI, which was used for PPI analysis by the Search Tool for the Retrieval of Interacting Genes/Proteins (STRING) database (version 9.1, http://string-db.org) [55]. *Brachypodium distachyon* L*.*, a model plant for economically important crop species including wheat and barley was selected as organism, and the PPI network with a confidence score of at least 0.700 was constructed [97, 98] and displayed using Cytoscape (version 3.0.2) software [99].

### Plant seedling cultivation and abiotic stress treatments

Seeds of Bd21 were kindly provided by Dr. John Vogel from the U.S. Department of Agriculture (USDA) Agricultural Research Service (ARS). The uniform seeds of standard diploid inbred line Bd21 were sterilized with 75% alcohol and 15% sodium hypochlorite, and then washed three times with sterile water. After sterilization, 500 g of seeds were germinated on water-filled filter paper for 3 days under complete darkness at 26 °C in three biological replicates. At the fourth day, seedlings were transferred to dedicated cultivation baskets with full-strength Hoagland solution (5 mM KNO_3_, 5 mM Ca(NO_3_)_2_, 2 mM MgSO_4_, 1 mM KH_2_PO_4_, 50 μM FeNa_2_(EDTA)_2_, 50 μM H_3_BO_3_, 10 μM MnC1_2_, 0.8 μM ZnSO_4_, 0.4 μM CuSO_4_, and 0.02 μM (NH_4_)_6_MoO_24_) in the greenhouse under a 16/8 h (light/dark) photocycle at 28/26 °C (day/night) condition with relative humidity of 70%. The nutrient solution was changed every 2–3 days. Five different organs and tissues (roots, stems, leaves, leaves tip and roots tip) were collected at three leaf stages in the control group as well as the developing seeds at 15 DPA. Meanwhile, seedlings were treated with the following conditions: salinity stress (200 mM NaCl), mild drought stress (20% (*w*/*v*) PEG 6000), heavy metal stress (300 μM CrCl_3_ and ZnSO_4_), hormone stress (100 μM ABA and SA, 10 μM IAA), hot stress (42 °C). Leaf and root samples of control seedlings were harvested at 0 h. The samples from heat stress were collected at 2 h, and those from other treated seedlings were harvested at 6, 12, 24, 48 h and recover 48 h. Each sample was collected from 10 plants with three biological replicates. All samples collected were immediately stored at − 80 °C prior to use.

### Total mRNA extraction and analysis of genes expression levels of *B. distachyon* by qRT-PCR

Total mRNA was extracted from the frozen samples collected using TRIzol Reagent (Invitrogen), and cDNA synthesis was performed using PrimeScript® RTReagent kit (TaKaRa, Shiga, Japan). All primers involved in the qRT-PCR process were designed using the online tool Primer3Plus (http://www.bioinformatics.nl/cgi-bin/primer3plus/primer3plus.cgi), and the specificity of the primers was examined by the corresponding dissociation curves and gel electrophoresis. Transcript levels were quantified using a CFX96 Real-Time PCR Detection System (Bio-Rad, Hercules, CA, USA) with the intercalating dye SYBR-green following the 2(−Delta Delta C(T)) method [100]. Ubiquitin (Bradi3g20790) was used as the reference gene, and qRT-PCR was performed according to Cao et al. [101], and three biological replicates were performed on each sample and Ct values were averaged.

## Additional files

Additional file 1: **Table S1**. The nomenclature, characteristics of *ARF* genes and their deduced proteins in seven representative plant species. **Table S2**. The parameters of conserved B3 and Auxin_resp domains from SMART and Pfam. **Table S3** Sequences of primers for subcellular localization. **Table S4**. Amino acid sites of functional divergence between groups of ARFs subfamily. **Table S5**. Adaptive selection analysis of *ARF* genes using site-specific models. **Table S6**. *cis*-element analysis of 1500 bp nucleotide sequences data upstream of the translation initiation codon of *ARF* genes. **Table S7**. Sequences of primers for qRT-PCR. (XLSX 262 kb)

Additional file 2: **Figure S1**. Exon-intron organization of *ARF* gene family. The bold yellow lines and gray lines represent exons and introns, respectively. The bold blue lines indicate the 5′ upstream region (left) and the 3′ downstream region (right). All *ARF* genes are divided into four categories according to the clades. The members of each clade are sorted by species. (JPG 2 mb)

Additional file 3: **Figure S2**. ARF proteins motifs schematic representation. The gray solid lines represent the corresponding ARF proteins and their length. The different-colored boxes represent different motifs and their position and order in individual ARF protein sequence. All *ARF* genes are divided into four categories according to clades and sorted according to the order of the phylogenetic tree. (JPG 2 mb)

Additional file 4: **Figure S3**. Composition of ARF protein motifs. The order of motifs in the Schematic representation was automatically generated by MEME according to scores. The symbol heights represent the relative frequency of each residue. (JPG 3 mb)

Additional file 5: **Figure S4**. Venn diagram of sites both in positive selection and functional divergence. In the Venn diagram, the green circle indicates the positive selection site, the yellow circle indicates the type I functional divergence site, and the blue circle indicates the type II functional divergence site. The intersections represent the sites that occurd between the two or among the three. (JPG 1 mb)

Additional file 6: **Figure S5**. Protein-protein interaction (PPI) network analysis of BdARF proteins. The figure shows the possible interactions between BdARFs and between BdARF and Aux/IAA family proteins. Red represents BdARF proteins with relatively close interactions, yellow represents BdARF proteins with a single interaction, and sky blue represents Aux/IAA family proteins (confidence score: 0.700). (JPG 711kb)

Additional file 7: **Figure S6**. qRT-PCR double standard curves of *BdARF* genes. The red standard curves represent the reference gene (S-adenosylmethionine decarboxylase gene), the blue standard curves represent the target genes. The double standard curves of different genes are indicated. A to S indicate *BdARF1* to *BdARF19* respectively. (JPG 3 mb)

Additional file 8: **Figure S7**. qRT-PCR dissociation curves of *BdARF* genes. The red standard curves represent the reference gene (S-adenosylmethionine decarboxylase gene), the blue standard curves represent the target genes. The dissociation curves of different genes are indicated. A to S indicate *BdARF1* to *BdARF19* respectively. (JPG 2 mb)

## Abbreviations

ABA: Abscisic acid
AD: Activation domain
ARF: Auxin response factor
Aux/IAA: Auxin/indole-3-acetic acid
AuxREs: Auxin response elements
CBF: C-repeat/dehydration-responsive element-binding factor
CDPK: Calcium Dependent Protein Kinase
CDS: Coding sequence
CTD: C-terminal dimerization domain
DAPI: 4^′^,6-diamidino-2-phenylindole
DBD: DNA-binding domain
DPA: Days post-anthesis
GFP: Green fluorescent protein
IAA: Indole-3-acetic acid;
MAPK: Mitogen activated protein kinase
MAPKK: MAPK kinase
MAPKKK: MAPKK kinase
MCMC: Markov Chain Monte Carlo
MEME: Multiple Em for Motif Elicitation
MR: Middle transcriptional regulatory region
MUSCLE: Multiple Sequence Comparison by Log-Expectation
MW: Molecular weight
PEG: Polyethylene glycol
pI: Isoelectric point
PPI: Protein-protein interaction
qRT-PCR: Quantitative real-time polymerase chain reaction
RD: Repression domain
RDs: Response to dehydration genes
ROS: Reactive oxygen species
SA: Salicylic acid
TIR1: Transport Inhibitor Resistant 1
