# Supplementary figures and images for: Genome-wide identification, molecular evolution, and expression analysis of auxin response factor (ARF) gene family in Brachypodium distachyon L
Source: BMC Plant Biol. 2018 Dec 6;18:336. doi: 10.1186/s12870-018-1559-z (PMC6282295; doi:10.1186/s12870-018-1559-z)

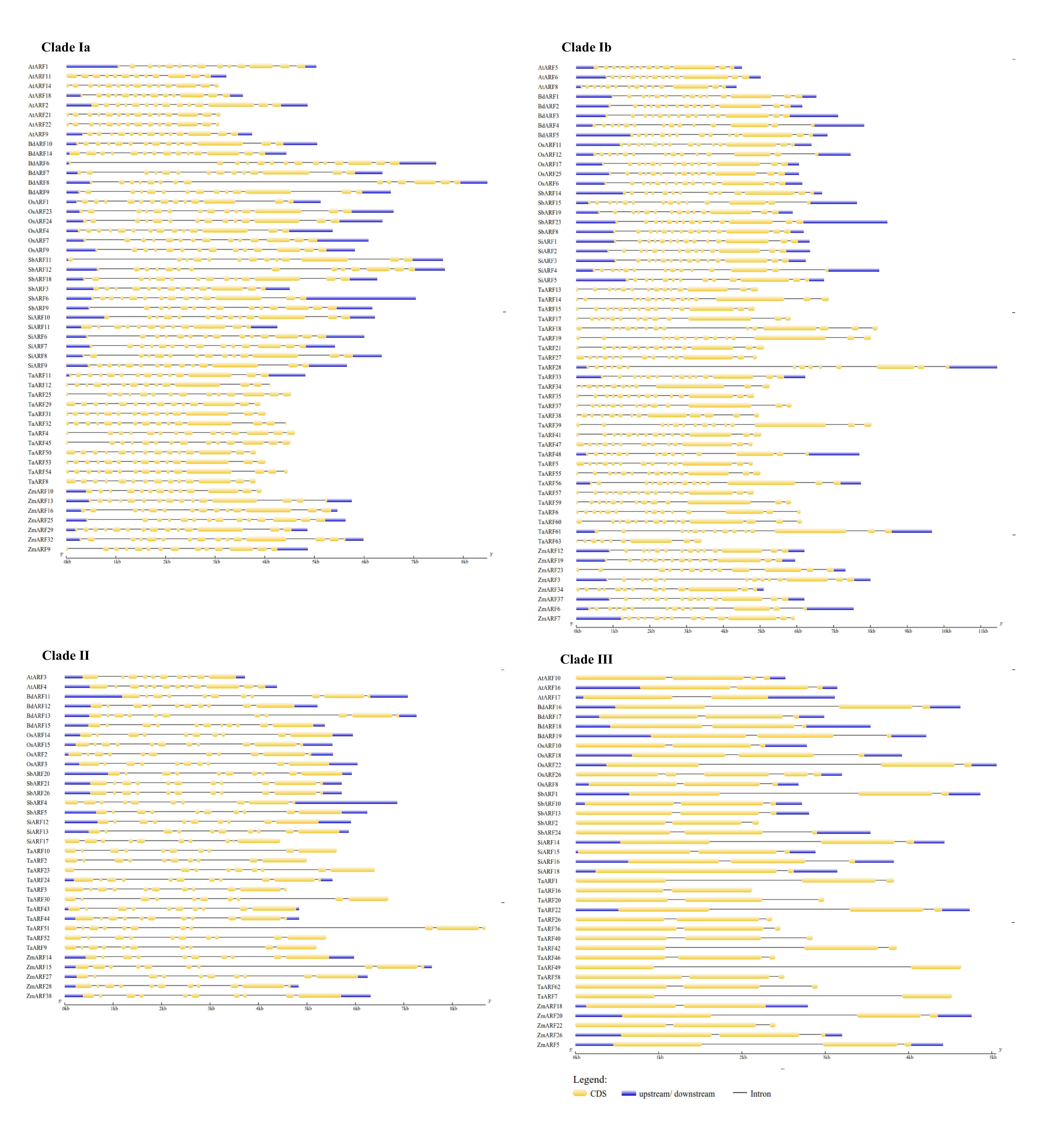

Supplement: Supplementary file 2 — Figure S1. Exon-intron organization of ARF gene family. The bold yellow lines and gray lines represent exons and introns, respectively. The bold blue lines indicate the 5′ upstream region (left) and the 3′ downstream region (right). All ARF genes are divided into four categories according to the clades. The members of each clade are sorted by species. (JPG 2 mb) [file 12870_2018_1559_MOESM2_ESM.jpg]

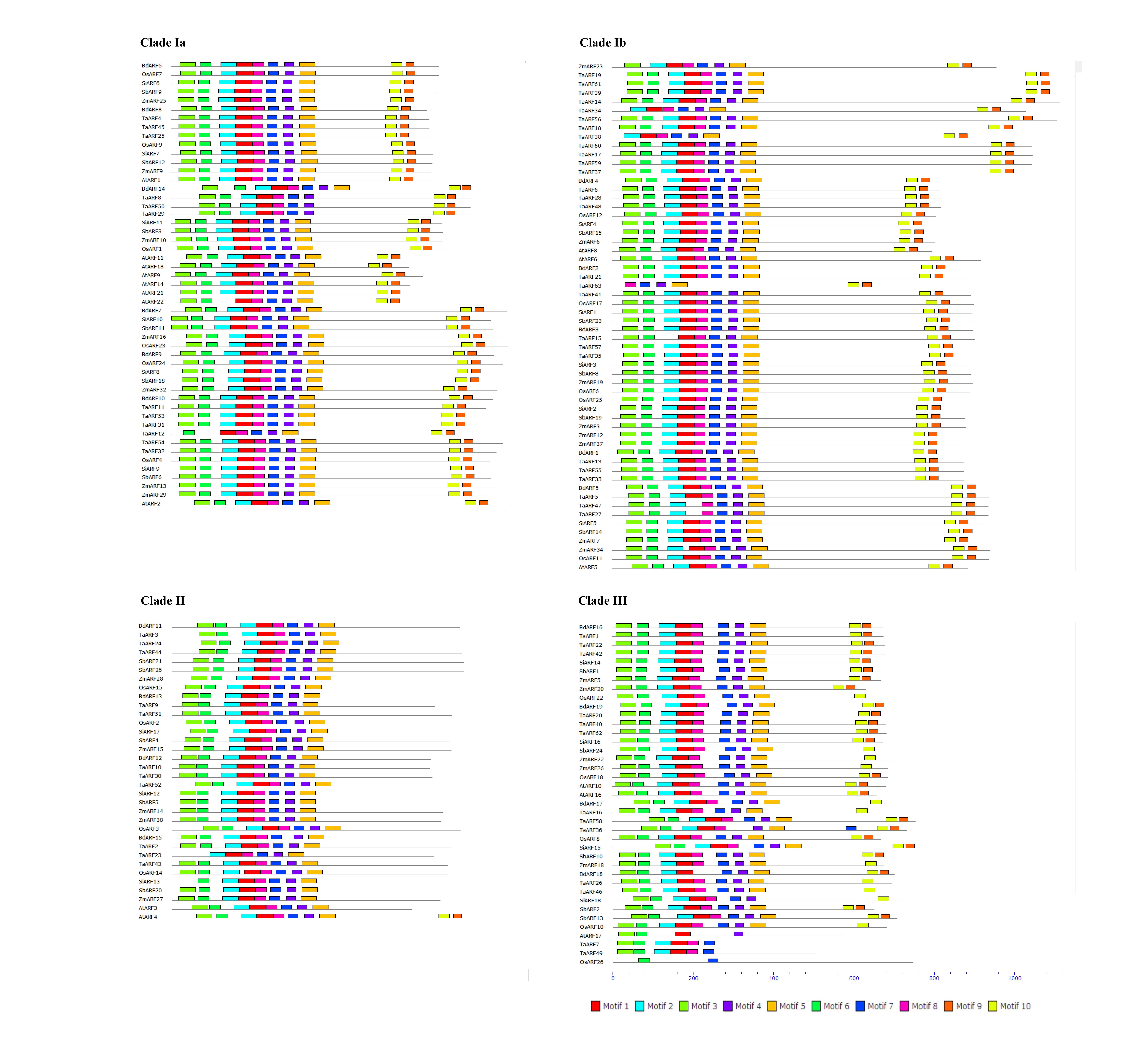

Supplement: Supplementary file 3 — Figure S2. ARF proteins motifs schematic representation. The gray solid lines represent the corresponding ARF proteins and their length. The different-colored boxes represent different motifs and their position and order in individual ARF protein sequence. All ARF genes are divided into four categories according to clades and sorted according to the order of the phylogenetic tree. (JPG 2 mb) [file 12870_2018_1559_MOESM3_ESM.jpg]

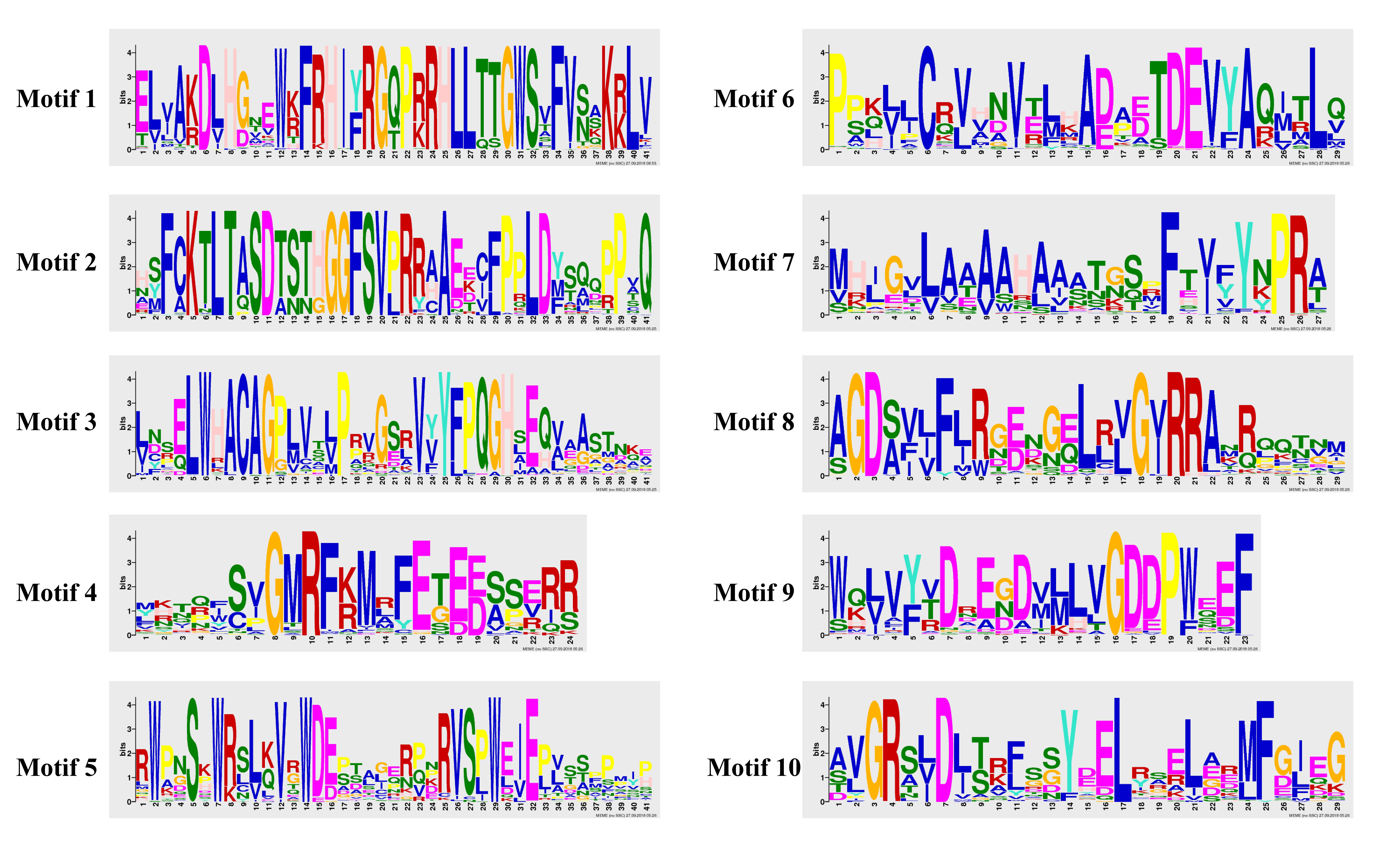

Supplement: Supplementary file 4 — Figure S3. Composition of ARF protein motifs. The order of motifs in the Schematic representation was automatically generated by MEME according to scores. The symbol heights represent the relative frequency of each residue. (JPG 3 mb) [file 12870_2018_1559_MOESM4_ESM.jpg]

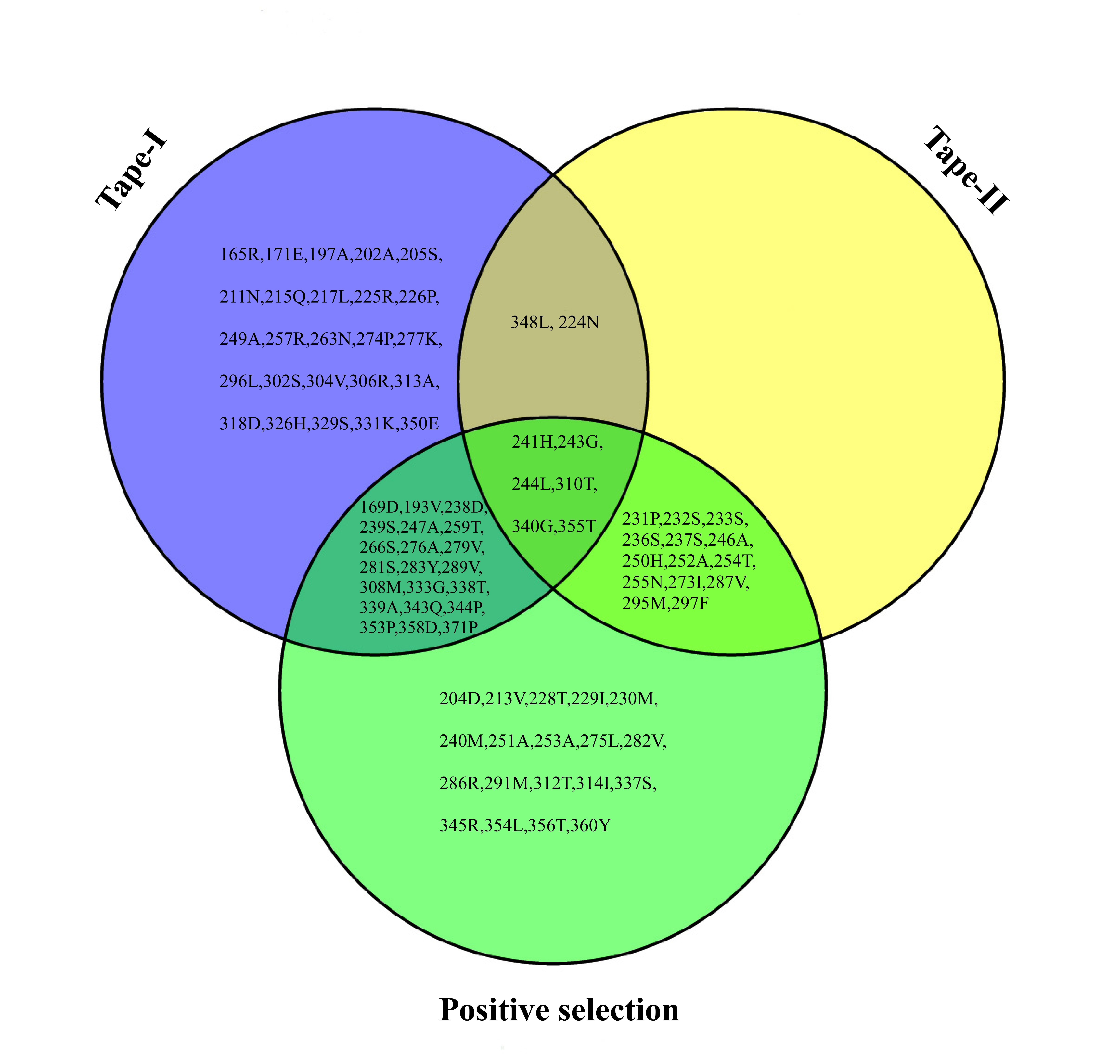

Supplement: Supplementary file 5 — Figure S4. Venn diagram of sites both in positive selection and functional divergence. In the Venn diagram, the green circle indicates the positive selection site, the yellow circle indicates the type I functional divergence site, and the blue circle indicates the type II functional divergence site. The intersections represent the sites that occurd between the two or among the three. (JPG 1 mb) [file 12870_2018_1559_MOESM5_ESM.jpg]

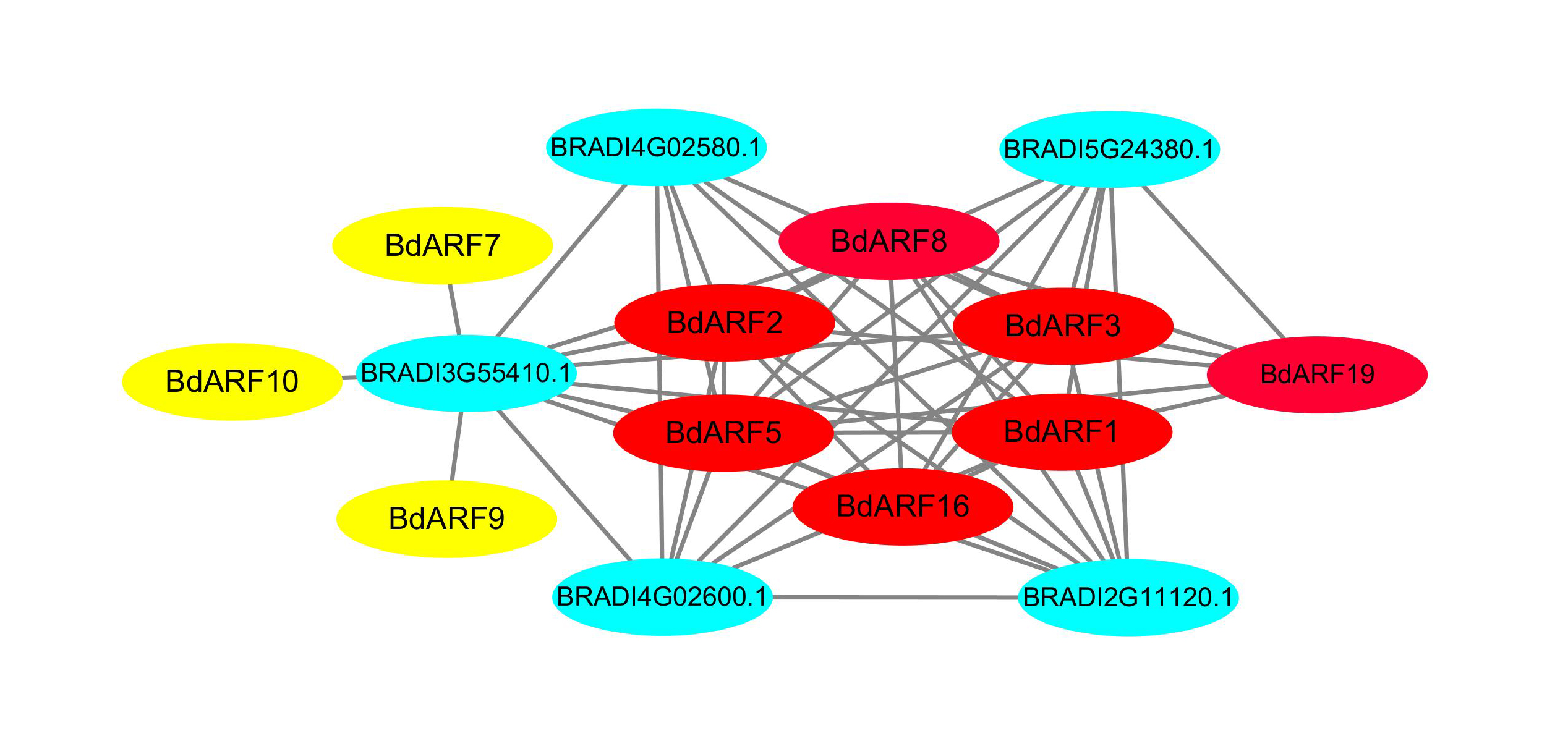

Supplement: Supplementary file 6 — Figure S5. Protein-protein interaction (PPI) network analysis of BdARF proteins. The figure shows the possible interactions between BdARFs and between BdARF and Aux/IAA family proteins. Red represents BdARF proteins with relatively close interactions, yellow represents BdARF proteins with a single interaction, and sky blue represents Aux/IAA family proteins (confidence score: 0.700). (JPG 711kb) [file 12870_2018_1559_MOESM6_ESM.jpg]

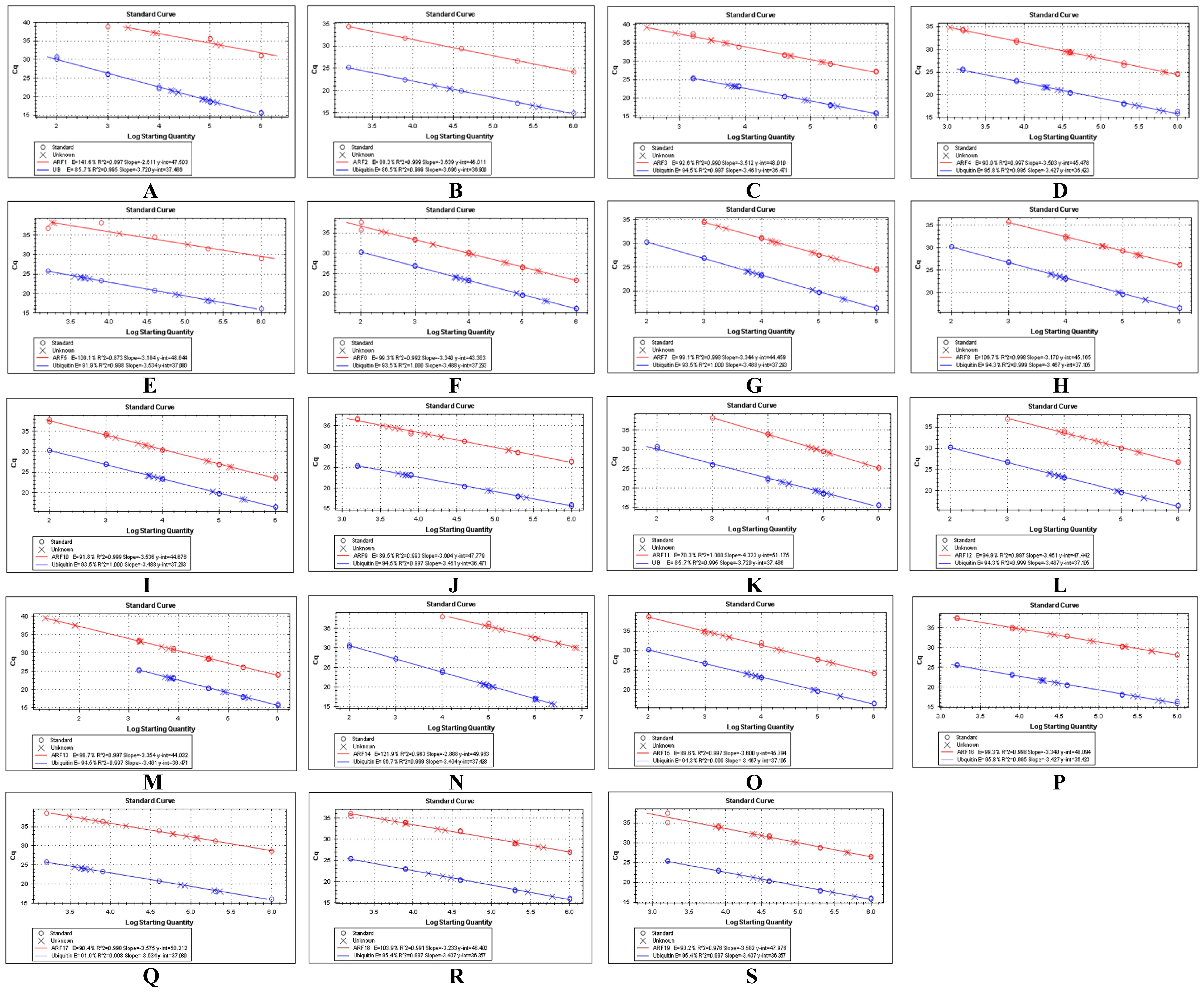

Supplement: Supplementary file 7 — Figure S6. qRT-PCR double standard curves of BdARF genes. The red standard curves represent the reference gene (S-adenosylmethionine decarboxylase gene), the blue standard curves represent the target genes. The double standard curves of different genes are indicated. A to S indicate BdARF1 to BdARF19 respectively. (JPG 3 mb) [file 12870_2018_1559_MOESM7_ESM.jpg]

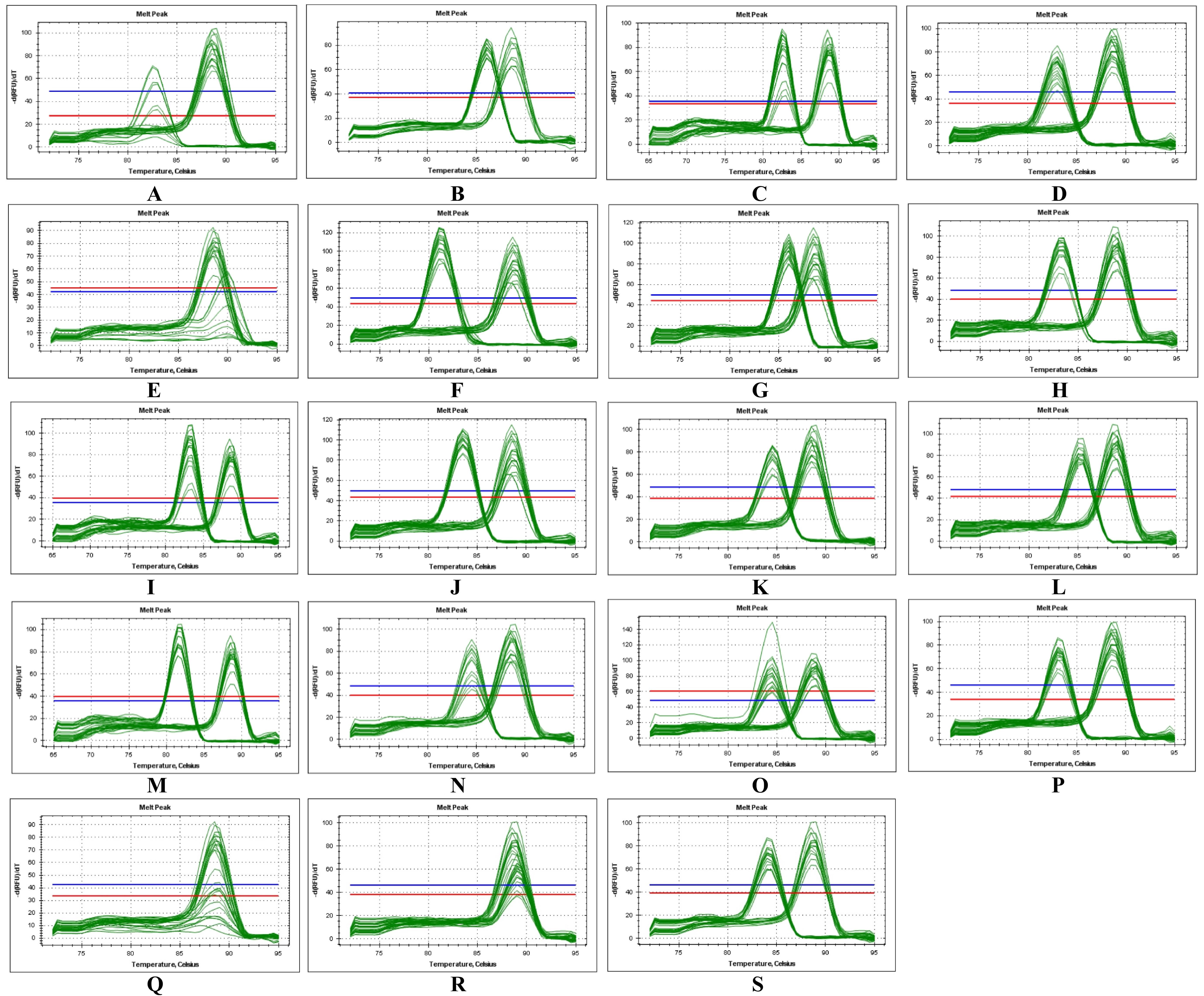

Supplement: Supplementary file 8 — Figure S7. qRT-PCR dissociation curves of BdARF genes. The red standard curves represent the reference gene (S-adenosylmethionine decarboxylase gene), the blue standard curves represent the target genes. The dissociation curves of different genes are indicated. A to S indicate BdARF1 to BdARF19 respectively. (JPG 2 mb) [file 12870_2018_1559_MOESM8_ESM.jpg]
